# Uric acid and cardiometabolic risk by gender in youth with type 1 diabetes

**DOI:** 10.1038/s41598-022-15484-0

**Published:** 2022-07-15

**Authors:** Procolo Di Bonito, Francesco Maria Rosanio, Maria Loredana Marcovecchio, Valentino Cherubini, Maurizio Delvecchio, Francesca Di Candia, Dario Iafusco, Angela Zanfardino, Brunella Iovane, Claudio Maffeis, Giulio Maltoni, Carlo Ripoli, Elvira Piccinno, Claudia Anita Piona, Maria Rossella Ricciardi, Riccardo Schiaffini, Adriana Franzese, Enza Mozzillo

**Affiliations:** 1 Department of Internal Medicine, “S. Maria Delle Grazie”, Pozzuoli Hospital, Naples, Italy; 2 Department of Translational Medical Science, Section of Pediatrics, Regional Center for Pediatric Diabetes, University of Naples Federico II, Via S. Pansini 5, 80131 Naples, Italy; 3 Department of Pediatrics, University of Cambridge, Cambridge, UK; 4 Department of Women’s and Children’s Health, Azienda Ospedaliero-Universitaria, Ospedale Riuniti Di Ancona, “G. Salesi” Hospital, Ancona, Italy; 5 Azienda Ospedaliero Universitaria Consorziale Policlinico Giovanni XXIII, Bari, Italy; 6 Department of Woman, Child and General and Specialized Surgery, University of Campania “Luigi Vanvitelli”, Naples, Italy; 7 Regional Diabetes Center, Children Hospital “Pietro Barilla”, University Hospital of Parma, Parma, Italy; 8 Section of Pediatric Diabetes and Metabolism, Department of Surgery, Dentistry, Pediatrics, and Gynecology, University and Azienda Ospedaliera Universitaria Integrata of Verona, Verona, Italy; 9 Department of Woman, Child and Urological Diseases, S. Orsola-Malpighi University Hospital, Bologna, Italy; 10 Pediatric Diabetology Unit, Pediatric and Microcytemia Department, AO Brotzu, Cagliari, Italy; 11 Diabetes Unit, Bambino Gesù Children’s Hospital, Rome, Italy

**Keywords:** Biomarkers, Nephrology, Risk factors

## Abstract

The aim of this study was to investigate the association between uric acid (UA) and cardiometabolic risk factors (CMRFs) by sex in youth with type 1 diabetes (T1D). Retrospective data collected from 1323 children and adolescents (5–18 years; 716 boys) with T1D recruited in 9 Italian Pediatric Diabetes Centers were analyzed. CMRFs included UA, HbA_1c_, blood pressure (BP), cholesterol (TC), HDL, triglycerides (TG), neutrophils (N) and lymphocytes (L) count, glomerular filtration rate (eGFR) (calculated using Schwartz-Lyon equation). In boys, we found a higher age, daily insulin dose, TG, TG/HDL ratio, TC/HDL ratio, systolic BP, N/L ratio and lower HDL, and eGFR across UA tertiles (*p* = 0.01–0.0001). Similar results were found in girls but not for TG and systolic BP. In boys, the odds ratio (OR) of high levels of TG/HDL ratio, TC/HDL ratio, BP and mildly reduced eGFR (MRGFR) increased for 0.5 mg/dL of UA. Instead, in girls an increased levels of 0.5 mg/dL of UA were associated with high OR of TC/HDL ratio, N/L ratio and MRGFR. Uric acid may represent a useful marker for identifying youth with T1D at high cardiometabolic risk, and this association appears to vary by sex.

## Introduction

Several cross-sectional and longitudinal studies have reported the independent role of uric acid (UA) as a predictor of cardiovascular morbidity, mortality and incident chronic kidney disease (CKD), in adult populations, especially with type 2 diabetes^1–4^. A robust association between UA and risk of incident cardiovascular events, mortality and an early onset of glomerular filtration rate (GFR) decline has also been reported in adults with type 1 diabetes (T1D)^5–7^. The mechanisms linking UA to cardio-renal disease risk are not fully elucidated yet. However, the effect of UA might be mediated by the activation of oxygen free radicals which in turn increase oxidative stress and inflammation^1,8^.

Several cross-sectional studies in pediatric populations, primarily performed in obese children, demonstrated that high UA levels are associated to metabolic syndrome and its individual components, nonalcoholic fatty liver disease, reduced estimated GFR (eGFR) and glucose dysmetabolism^9–11^.

In contrast, in adolescents with T1D, the relationship between UA levels and cardiometabolic risk factors (CMRFs) is controversial and little explored^12,13^. This is an important issue which deserves clarification since it is well known that cardiovascular prevention should start as early as possible, not only in children with obesity and type 2 diabetes but also in those with T1D^14–16^. Furthermore, a potential sex-related difference in the relationship between UA and CMRF has not been well explored so far^12,13^. Therefore, the aim of the present study was to evaluate the cardiometabolic risk (CMR) profile associated with UA levels in a large sample of children and adolescents with T1D. In addition, we assessed whether the UA-related CMR profile is different among the two sexes.

## Results

The study population included 1323 individuals with T1D, 716 boys and 607 girls, with a mean (± SD) age of 13.5 ± 3.1 years. The clinical and biochemical characteristics of the study population are summarized in Table 1. There were no missing data. In boys, statistically significant differences were found across UA tertiles for age, BMI, daily insulin dose, HDL, TG, TG/HDL ratio, TC/HDL ratio, N/L ratio, systolic BP, eGFR, across tertiles of UA (*p* = 0.01–0.0001) (Table 2).

**Table 1:** Anthropometric and biochemical features of the study population by sex.

	All	Boys	Girls	***p* value**
n	1323	716	607	
Age (years)	13.5 ± 3.1	13.6 ± 3.0	13.4 ± 3.2	0.141
Prepubertal stage, n (%)	59 (4.5)	29 (4.1)	30 (4.9)	0.433
BMI (kg/m^2^)	20.7 ± 3.7	20.7 ± 3.7	20.8 ± 3.7	0.618
BMI-SDS	0.05 ± 1.0	− 0.02 ± 1.0	0.13 ± 1.0	0.005
Diabetes duration (years)	6.5 ± 3.3	6.5 ± 3.2	6.4 ± 3.3	0.531
Autoimmune diseases (%)	299 (22.6)	156 (22)	143 (23.6)	0.443
Insulin dose (IU/kg/day)	1.1 ± 2.0	1.0 ± 0.6	1.2 ± 3.0	0.020
HbA_1c_ (%)	7.8 ± 1.2	7.8 ± 1.2	7.8 ± 1.2	0.928
HbA1c (mmol/mol)	62.0 ± 13.0	62.0 ± 12.8	62.1 ± 13.3	0.928
CSII, n (%)	460 (35)	245 (34)	215 (35)	0.647
Albuminuria, (%)	90 (6.8)	37 (5.2)	53 (8.7)	0.010
Total cholesterol (mg/dL)	165.0 ± 31.4	165.5 ± 31.7	164.3 ± 31.1	0.511
HDL (mg/dL)	59.2 ± 12.8	59.5 ± 12.4	58.8 ± 13.2	0.372
Triglycerides (mg/dL)	67.0 ± 37.8	67.1 ± 40.0	67.0 ± 35.1	0.478
TG/HDL ratio	1.2 ± 0.9	1.2 ± 0.9	1.2 ± 0.8	0.301
TC/HDL ratio (mg/dL)	2.9 ± 0.7	2.9 ± 0.7	2.9 ± 0.7	0.508
SPISE index	10.2 ± 2.7	10.3 ± 2.7	10.2 ± 2.7	0.315
N/L ratio	1.5 ± 0.8	1.6 ± 0.8	1.5 ± 0.8	0.213
Systolic BP (mmHg)	109.0 ± 11.8	109.3 ± 12.1	108.6 ± 11.4	0.268
Diastolic BP (mmHg)	67.4 ± 7.9	67.3 ± 8.1	67.6 ± 7.6	0.458
eGFR (mL/min/1.73 m^2^)	103.7 ± 26.0	106.1 ± 25.9	100.9 ± 25.9	< 0.0001
Uric acid (mg/dL)	3.50 ± 1.0	3.53 ± 1.0	3.47 ± 1.0	0.274

Data are expressed as mean ± SD, n (%). *p* values are for differences between boys and girls.

HbA_1c_: glycosylated hemoglobin, CSII: continuous subcutaneous insulin infusion. MDI: multiple doses of insulin. IU/Kg/day: insulin dose pro Kg Day. TG/HDL ratio: triglycerides to HDL ratio. TC/HDL ratio: Cholesterol to HDL ratio. SPISE: single-point insulin sensitivity estimator N/L ratio: Neutrophil/Lymphocyte ratio. BP: blood pressure. eGFR: estimated glomerular filtration rate. HbA_1c_: glycosylated hemoglobin, CSII: continuous subcutaneous insulin infusion. MDI: multiple doses of insulin. IU/Kg/day: insulin dose pro Kg Day. TG/HDL ratio: triglycerides to HDL ratio. TC/HDL ratio: Cholesterol to HDL ratio. SPISE: single-point insulin sensitivity estimator N/L ratio: Neutrophil/Lymphocyte ratio. BP: blood pressure. eGFR: estimated glomerular filtration rate.

**Table 2:** Anthropometric and biochemical features in boys according to tertiles of uric acid.

UA (mg/dl)	Uric acid tertiles			p value
	< 3.0	≥ 3.0 < 3.9	≥ 3.9	
n = 716	216	254	246	
Age (years)	13.2 ± 2.8	13.8 ± 3.1	13.9 ± 3.0	0.028
Prepubertal stage, n (%)	8 (3.7)	10 (3.9)	11 (4.5)	0.911
BMI (kg/m^2^)	19.8 ± 3.1	20.9 ± 3.9	21.3 ± 3.8	< 0.0001
BMI-SDS	− 0.20 ± 0.9	0.02 ± 1.0	0.09 ± 1.1	0.004
Diabetes duration (years)	6.3 ± 3.2	6.7 ± 3.3	6.6 ± 3.2	0.346
Autoimmune diseases	46 (21)	55 (22)	55 (22)	0.961
Insulin dose (IU/Kg/day)	1.0 ± 0.6	1.0 ± 0.6	1.1 ± 0.6	0.022
HbA_1c_ (%)	7.9 ± 1.2	7.8 ± 1.2	7.7 ± 1.1	0.308
HbA_1c_ (mmol/mol)	62.8 ± 13.0	62.1 ± 13.0	61.1 ± 12.3	0.308
CSII, n (%)	75 (35)	93 (37)	77 (31)	0.449
Albuminuria, (%)	9 (4.2)	14 (5.5)	14 (5.7)	0.726
Total cholesterol (mg/dL)	166.3 ± 31.8	168.1 ± 31.9	162.0 ± 30.8	0.089
HDL (mg/dL)	61.9 ± 11.9	59.9 ± 12.8	56.9 ± 12.2	< 0.0001
Triglycerides (mg/dL)	60.0 ± 27.8	68.1 ± 44.7	72.3 ± 43.1	0.001
TG/HDL ratio	1.0 ± 0.6	1.2 ± 0.9	1.4 ± 1.1	< 0.0001
TC/HDL ratio (mg/dL)	2.8 ± 0.7	2.9 ± 0.7	2.9 ± 0.7	0.006
N/L ratio	1.5 ± 0.8	1.5 ± 0.7	1.7 ± 1.0	0.015
Systolic BP (mmHg)	106.9 ± 11.3	109.0 ± 12.2	111.6 ± 12.2	< 0.0001
Diastolic BP (mmHg)	66.9 ± 7.5	66.7 ± 8.3	68.2 ± 8.2	0.096
eGFR (mL/min/1.73 m^2^)	115.1 ± 27.4	107.5 ± 24.4	96.9 ± 23.1	< 0.0001

Data are expressed as mean ± SD, n (%). *p* values are for trends across tertiles.

HbA_1c_: glycosylated hemoglobin, CSII: continuous subcutaneous insulin infusion. MDI: multiple doses of insulin. IU/Kg/day: insulin dose pro Kg Day. TG/HDL ratio: triglycerides to HDL ratio. TC/HDL ratio: Cholesterol to HDL ratio. SPISE: single-point insulin sensitivity estimator N/L ratio: Neutrophil/Lymphocyte ratio. BP: blood pressure. eGFR: estimated glomerular filtration rate.

In girls, statistical differences between tertiles of UA were observed for age, daily insulin dose, HDL, TG/HDL ratio, TC/HDL ratio, N/L ratio and eGFR (*p* = 0.04–0.0001) (Table 3).

**Table 3:** Anthropometric and biochemical features in girls according to tertiles of uric acid.

UA (mg/dl)	Uric acid tertiles			p value
	< 3.0	≥ 3.0 < 3.8	≥ 3.8	
n = 607	187	217	203	
Age (years)	13.0 ± 3.3	13.4 ± 3.2	13.8 ± 3.0	0.041
Prepubertal stage, n (%)	12 (6.4)	11 (5.1)	7 (3.4)	0.399
BMI (kg/m^2^)	20.8 ± 3.8	20.6 ± 3.5	21.0 ± 3.9	0.595
BMI-SDS	0.19 ± 1.0	0.10 ± 1.0	0.11 ± 1.0	0.616
Diabetes duration (years)	6.3 ± 3.2	6.5 ± 3.3	6.5 ± 3.3	0.787
Autoimmune diseases	39 (21)	44 (20)	50 (30)	0.047
Insulin dose (IU/kg/day)	1.2 ± 2.1	1.3 ± 4.2	1.2 ± 1.9	0.040
HbA_1c_ (%)	8.0 ± 1.3	7.8 ± 1.2	7.7 ± 1.2	0.085
HbA1c (mmol/mol)	63.4 ± 14.0	62.3 ± 12.8	60.1 ± 13.0	0.085
CSII, n (%)	66 (35)	79 (36)	70 (34)	0.918
Albuminuria, (%)	16 (8.6)	20 (9.2)	17 (8.4)	0.949
Total cholesterol (mg/dL)	166.0 ± 32.2	164.7 ± 30.9	162.4 ± 30.1	0.515
HDL (mg/dL)	62.1 ± 12.9	58.2 ± 12.8	57.5 ± 12.8	0.001
Triglycerides (mg/dL)	64.1 ± 32.5	66.4 ± 35.6	70.3 ± 36.8	0.226
TG/HDL ratio	1.1 ± 0.7	1.2 ± 0.7	1.3 ± 0.9	0.012
TC/HDL ratio (mg/dL)	2.8 ± 0.7	2.9 ± 0.7	2.9 ± 0.8	0.019
N/L ratio	1.4 ± 0.7	1.5 ± 0.8	1.6 ± 0.8	0.031
Systolic BP (mmHg)	107.5 ± 11.9	108.7 ± 12.1	109.4 ± 10.2	0.247
Diastolic BP (mmHg)	68.4 ± 7.8	67.4 ± 7.8	67.0 ± 7.4	0.179
eGFR (mL/min/1.73 m^2^)	107.9 ± 28.9	101.1 ± 26.5	94.2 ± 20.1	< 0.0001

Data are expressed as mean ± SD, n (%). *p* values are for trends across tertiles.

HbA_1c_: glycosylated hemoglobin, CSII: continuous subcutaneous insulin infusion. MDI: multiple doses of insulin. IU/Kg/day: insulin dose pro Kg Day. TG/HDL ratio: triglycerides to HDL ratio. Chol/HDL ratio: Cholesterol to HDL ratio. SPISE: single-point insulin sensitivity estimator N/L ratio: Neutrophil/Lymphocyte ratio. BP: blood pressure. eGFR: estimated glomerular filtration rate.

**Figure 1:**
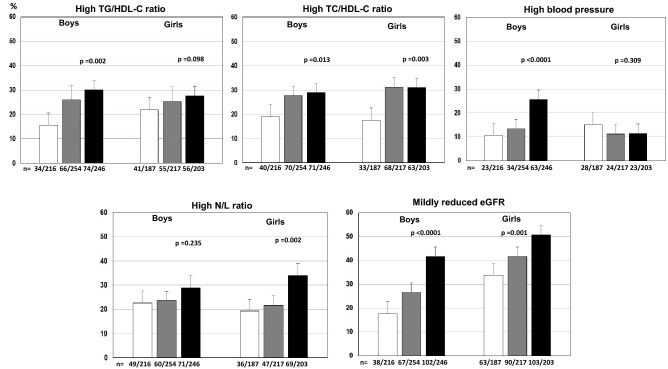
Proportions (mean and 95% CI) age-adjusted of youth with abnormal CMRFs across tertiles of UA both in boys and girls.

The age-adjusted proportions (mean and 95% CI) of youth with abnormal CMRFs across tertiles of UA both in boys and girls are shown in Fig. 1. A statistically significant increase across UA tertiles was found in both sexes for high TC/HDL ratio, and MRGFR. In boys, a higher prevalence of high blood pressure was found, whereas in the female group the prevalence of high N/L ratio varied across UA tertiles.

In boys, the odds ratio of high levels of TG/HDL ratio, TC/HDL ratio, BP and MRGFR increased for 0.5 mg/dL of UA; instead, in girls a 0.5 mg/dL increase in UA was associated with high odds ratio of TC/HDL ratio, N/L ratio and MRGFR (Table 4).

**Table 4:** Odds ratio (95%Cl) of cardiometabolic risk factors for 0.5 mg/dL of increased level in uric acid by gender.

	Boys	p value**	Girls	p value***
High TG/HDL ratio	2.00 (1.37–2.90)	< 0.0001	1.32 (0.89–1.97)	0.172
High TC/HDL ratio	1.66 (1.16–2.39)	0.006	1.69 (1.14–2.50)	0.009
High N/L ratio	1.31 (0.91–1.88)	0.142	1.91 (1.29–2.82)	0.001
High BP	2.37 (1.56–3.60)	< 0.0001	0.64 (0.37–1.10)	0.110
MRGFR	3.52 (2.37–5.21)	< 0.0001	1.73 (1.12–2.67)	0.014

**p value adjusted for centers, age, BMI-SDS, insulin dose/Kg/Die.

***p value adjusted for centers, age, insulin dose/Kg/Die.

## Discussion

Our study showed a potential association between UA and CMRFs in children and adolescents with T1D. This association suggests that UA could be a potential marker of cardiometabolic risk in this population. Furthermore, our study showed that the CMR risk profile associated with UA is worse in males than in females.

High levels of UA have been reported in adults with hypertension, obesity and metabolic syndrome^1^. Furthermore, prospective studies have shown the usefulness of UA in predicting incident cardio-renal events both in individuals with T1D and type 2 diabetes^2–7^.

In children, recent studies have shown a strong association between UA, nonalcoholic fatty liver disease mildly reduced eGFR and glucose dysmetabolism^10,11^. These studies have been performed in obese youth, whereas few and conflicting observations have been reported in youth with T1D^12,13^. Indeed, Lytvyn et al. could not detect any relationship between UA and cardio-renal abnormalities in their study conducted in small sample of 180 adolescents with T1D^13^. In contrast, Słomiński et al. demonstrated a positive association between UA and both nephropathy and subclinical inflammatory marker concentrations in boys but not in girls with T1D^12^. Differences findings between studies might be related to sample size, age range and other specific characteristics of the study populations, such as ethnicity.

In our study, we performed a comprehensive analysis including markers of low grade-inflammation and UA levels separately in the two sexes. To the best of our knowledge, our study demonstrates for the first time a robust association between CMRFs and UA in children and adolescents with T1D, which was more pronounced in boys than in girls.

Uric acid levels are higher in adult males than females, as well in obese boys than girls^1,10,11^. This difference is likely due to a distinct roles of sex hormones and a higher muscle mass in males^17^*.* In our study population, we did not observe any difference in UA levels between the two sexes, and this could be related most of participants being normal weight as well as to the specific age range.

In childhood, high values of UA are influenced by ethnicity, age and sex, so a universal definition of abnormal UA levels is lacking. In non-obese youth values above 6 or 7 mg/dL in boys and 5 or 6 mg/dL in girls were previously considered “elevated”^18,19^.

In children and adolescents with T1D levels of UA are lower than healthy individuals^20^. This result could lead to undermining the usefulness of assessing UA in clinical practice in youth with T1D. Despite this, our study showed a robust negative association between 'high normal' levels of UA and eGFR, in line with our previous studies^21,22^ and extends a positive association between UA and different CMRFs in young people with T1D. Interestingly, the results differed by sex, with boys having an increased risk for high TG/HDL, TC/HDL ratio, high BP and MRGFR, whereas girls showed a high risk of abnormal TC/HDL ratio, N/L ratio and MRGFR.

The different impact of UA in males vs females is still undefined. In our sample, we confirm in boys an association between high BP and high levels of UA as reported in a Caucasian sample of obese youth^23^. This suggests the link between UA, even in a high normal range, and elevated BP levels that may be useful to identify boys with T1D at high risk of hypertension.

In the present study, an association between the TG/HDL ratio and UA in boys was also detected. Insulin resistance may represent the link between the TG/HDL ratio and UA.

A further novel finding from our study is the potential association between high levels of UA and high TC/HDL ratio. This lipid ratio is considered a marker of atherogenic dyslipidemia^24^ and a more sensitive predictor of cardiovascular events in adults than total cholesterol^25^. Interestingly, the association between UA and high TC/HDL ratio is shared by the two sexes, independently of BMI. This further supports the usefulness of UA as marker of early atherosclerosis in both sexes.

In adults and obese children, the relationship between UA and MRGFR is well established^1,10^, whereas this association has been little explored in T1D, especially in young people^12,13^. The strong associations we found between UA and MRGFR in both boys and girls is in line with our previous reports^21,22^. The strength of this association sustains the evaluation of UA in clinical practice to identify youth with T1D at risk of eGFR decline in both sexes. Our results might have influenced by the specific formula used to calculate eGFR. At present, there is no consensus regarding the best method for estimating GFR in children and adolescents with T1D. We used a recently validated formula for the pediatric population, which relies on the use of creatinine and it is easily implemented into clinical practice^26^.

Of particular interest is the link between a high N/L ratio, as surrogate of low-grade inflammation, and high levels of UA in girls. The N/L ratio has been recently studied in several conditions, such as cardiac, vascular, and kidney disease where the low-grade inflammation was potentially involved^27^. In particular, recent studies demonstrated that a higher N/L ratio represents a useful marker to identify diabetic kidney disease^28^. In our sample we observed a robust association between N/L ratio and high levels of UA in girls, but not in boys. This association may be supported by higher percentage of girls with concomitant autoimmune diseases as compared to boys. This finding contrasts with those form Słomiński et al.^12^, who reported a positive association between UA and subclinical inflammatory marker concentrations only in boys but not in girls. The reasons for this discrepancy are unknown and warrant further study.

Some limitations of the present study need to be acknowledged. Firstly, being a retrospective multicenter, clinic-based study, data were collected and analyzed across different centers. However, all laboratories were standardized and used similar methods that were aligned. In addition, it is important to acknowledge that the study methods reflect real-word data collection and therefore closely reflect what can be implemented in daily clinical practice. The study population lacked diversity in terms of ethnicity, and this may limit the generalization of the study findings. However, despite these limitations, the present study, based on a large sample size and data collection, highlighted an important role of UA as potential early marker of cardiometabolic risk.

In conclusion, our study suggests that UA levels in youth with T1D could be useful to identify those at higher cardio-metabolic risk, although sex-related differences need to be taken into account. If confirmed by future prospective studies, the present findings could lead towards the implementation of UA as part of the set of investigations required in all youth with T1D from early stages of the disease to support prediction and prevention of cardio-metabolic complications.

## Methods

This multicenter retrospective cross-sectional study including Caucasian children and adolescents with T1D consecutively recruited within 16 months from 1 January 2019 to 30 April 2020 in 9 Italian Pediatric Diabetes Centers that are part of the Diabetes Study Group of the Italian Society for Pediatric Endocrinology and Diabetology.

Medical history, biochemical and clinical data were retrieved from clinical records. Inclusion criteria were: availability of data regarding UA, diagnosis of T1D (at least 1 positive autoantibody), diabetes duration > 1 year, age 5–18 years, availability of anthropometric and biochemical data. Finally were analyzed records of 1323 youth. The study protocol was approved by the Ethics Committees of the participating Centers (Federico II University of Naples, coordinating center; Ancona; Bari; Campania Vanvitelli University of Naples; Parma; Verona; Bologna; Cagliari; Roma) and was conducted in accordance with the declaration of Helsinki and good clinical practice guidelines. Informed written consent was obtained from all the parents and/or patients before their inclusion in the study. The clinical and biochemical parameters were anonymously entered in a database using an alphanumeric and progressive code.

### Measurements

For all study participants, the following data were recorded by each of the nine participating centers: age, sex, height, weight, body mass index (BMI), blood pressure (BP), serum creatinine (Scr), UA, HbA_1c_, total cholesterol (TC), triglycerides (TG), HDL, Neutrophil (N) and Lymphocyte (L) counts, albuminuria and insulin regimen (multiple daily injections vs continuous subcutaneous insulin infusion) and doses (IU/kg/day). Weight and height were collected by a single trained operator in each center. BMI-SDS was calculated based on the Italian BMI normative data^29^.

In all centers HbA_1c_ was assessed by High-Performance Liquid Chromatography; TC, TG and HDL were determined by enzymatic methods; N and L counts were measured using an automated analyzer.

Scr was analyzed in 4 centers by enzymatic method and in 5 centers using Jaffé IDMS traceable method, while UA by the uricase method in all centers^30^. The relationship between Scr, age, sex, BMI and enzymatic method was explored by a linear regression analysis using Scr as dependent variable and age, sex, BMI and enzymatic method as covariate. We obtained a B coefficient for enzymatic method that was subtracted from the value of Scr assessed by Jaffé method. The value of Scr assessed by Jaffé method resulted identical to that obtained with enzymatic method as elsewhere described^21^.

The following parameters: TG to HDL (TG/HDL), TC to HDL (TC/HDL) and Neutrophil to Lymphocyte ratio (N/L) were calculated^31^.

Estimated glomerular filtration rate (eGFR) was calculated using Schwartz-Lyon equation^31^:

$${\text{eGFR}} = [\text{k} \times {\text{height (cm)/Scr (mg/dL)}}], \text{k} = 36.5 {\text{in males aged}} > 13 {\text{years}}, 32.5 {\text{in others}}.$$

Evaluation of albuminuria was determined in 4 centers (n = 697) as albumin to creatinine ratio (ACR) (using an enzymatic method for assessment of Scr) on first-morning non-orthostatic urine samples, while in 5 centers (n = 626) as albumin excretion rate (AER) was assessed as timed urine collection, as recommended by the kidney disease: Improving Global Outcomes (KDIGO) clinical practice guideline^32^. Albuminuria was defined as normal to mildly increased (ACR < 30 mg/g; AER < 30 mg/d); moderately increased (ACR 30–300 mg/g; AER 30–300 mg/d); severely increased (ACR > 300 mg/g; AER > 300 mg/d)^32^. The presence of albuminuria was defined by positivity in at least two different measurements.

BP was measured following the recommendations by the European Society of Hypertension^33^. BP was measured, after 5 min of resting in a quiet room, using an appropriate sized arm cuff on the right arm and an aneroid sphygmomanometer. Three measurements were obtained every 2 min and the mean of the last two values were used in the analyses.

All blood samples for biochemical analyses were collected in each center after 12 h of fasting. Although laboratory analyses were performed in different laboratories, these are all part of the Italian National Health System and are certified according to International Standards ISO 9000 (www.iso9000.it/)^34^.

### Definitions

Presence of puberty in all patients was identified as breast development in girls and testicular growth to at least 4 mL in volume in boys; pre-puberty in case of absence of any sign of puberty^35^. Autoimmune diseases were defined by the presence of autoimmune thyroiditis and/or celiac disease. Albuminuria (microalbuminuria) was defined as moderately increased (ACR 30–300 mg/g; AER 30–300 mg/d) or severely increased (ACR > 300 mg/g, > 300 mg/d) (macroalbuminuria)^32^. The presence of albuminuria was defined by positivity in at least two different measurements^36^.

High BP was defined using criteria proposed by the American Academy of Pediatrics: BP ≥ 95^th^ percentile for age, sex and height in children aged less than 13 years or BP ≥ 130/80 mmHg in adolescents (age ≥ 13 years)^37^.

High TG/HDL ratio, TC/HDL ratio and N/L ratio were defined by the 75^th^ percentile of the study population. Mildly reduced eGFR was defined by a value between 60–89 mL/min/1.73 m^2^^32^.

### Statistics

Data are expressed as mean ± standard deviation, median and interquartile range, or absolute and relative frequencies, unless otherwise stated. Given the skewed distribution of HbA_1c_, TG, TG/HDL ratio, TC/HDL ratio, N/L ratio and insulin dose, the statistical analysis of these variables was applied after log transformation and back transformation to natural units to allow presentation in the text and tables. Between groups differences in continuous variables were assessed with Student’s *t*-test. Analysis of variance (ANOVA) was applied to evaluate differences across tertiles of UA. The *χ*^2^ and Fisher’s exact tests, as appropriate, were used to compare categorical variables. Exact tests were performed using the Monte Carlo method. To evaluate the association (Odds ratio, 95% CI) between the CMRFs and UA, a logistic regression analysis was performed using the high levels of each CMRF as dependent variable and centers, age, insulin dose (IU/kg/day), BMI-SDS and 0.5 mg/dL of UA as covariates. Statistical analysis was performed using the IBM SPSS Statistics for Windows, Version 27. A *p*-value < 0.05 was considered statistically significant.

### Ethics approval

The study protocol was approved by the ethics committees of all participating centers and was conducted in accordance with the declaration of Helsinki and good clinical practice guidelines.

## Data Availability

The datasets generated during and/or analyzed during the current study are available from the corresponding author on reasonable request.
